# Perceived quality of life among caregivers of children with a childhood-onset dystrophinopathy: a double ABCX model of caregiver stressors and perceived resources

**DOI:** 10.1186/s12955-017-0612-1

**Published:** 2017-02-10

**Authors:** Natalia Frishman, Kristin Caspers Conway, Jennifer Andrews, Jacob Oleson, Katherine Mathews, Emma Ciafaloni, Joyce Oleszek, Molly Lamb, Dennis Matthews, Pangaja Paramsothy, Lowell McKirgan, Paul Romitti

**Affiliations:** 1 Department of Epidemiology, The University of Iowa, Iowa City, USA; 2 Department of Pediatrics, The University of Arizona, Tucson, USA; 3 Department of Biostatistics, The University of Iowa, Iowa City, USA; 4 Departments of Pediatrics and Neurology, The University of Iowa, Iowa City, USA; 5 Departments of Neurology and Pediatrics, University of Rochester Medical Center, Rochester, USA; 6 Department of Physical Medicine and Rehabilitation, University of Colorado and Children’s Hospital Colorado, Aurora, USA; 7 Department of Epidemiology, Colorado School of Public Health, Aurora, USA; 8 National Center on Birth Defects and Developmental Disabilities, Centers for Disease Control and Prevention, Atlanta, USA; 9 Departments of Epidemiology and Biostatistics and Interdisciplinary Program in Toxicology, The University of Iowa, College of Public Health, S416 CPHB, 145 N Riverside Dr, Iowa City, IA 52242 USA; 10 Present address: General Dynamics Information Technology, Coralville, IA USA

**Keywords:** Becker muscular dystrophy, Caregivers, Duchenne muscular dystrophy, Dystrophinopathy, Muscular dystrophies, Quality of life

## Abstract

**Background:**

Duchenne and Becker muscular dystrophies, collectively referred to as dystrophinopathies, are recessive X-linked disorders characterized by progressive muscle weakness and ultimately cardiac and respiratory failure. Immediate family members are often primary caregivers of individuals with a dystrophinopathy.

**Methods:**

We explored the impact of this role by inviting primary caregivers (*n* = 209) of males diagnosed with childhood-onset dystrophinopathy who were identified by the Muscular Dystrophy Surveillance, Tracking, and Research Network (MD STAR*net*) to complete a mailed questionnaire measuring perceived social support and stress, spirituality, and family quality of life (FQoL). Bivariate and multivariate analyses examined associations between study variables using the Double ABCX model as an analytic framework.

**Results:**

Higher stressor pile-up was associated with lower perceived social support (*r* = -0.29, *p* < .001), availability of supportive family (*r* = -0.30, *p* < .001) or non-family (*r* = -0.19, *p* < .01) relationships, and higher perceived stress (*r* = 0.33, *p* < .001); but not with spirituality (*r* = -0.14, *p* > 0.05). FQoL was positively associated with all support measures (correlations ranged from: 0.25 to 0.58, *p*-values 0.01–0.001) and negatively associated with perceived stress and control (*r* = -0.49, *p* < .001). The association between stressor pile-up and FQoL was completely mediated through global perceived social support, supportive family relationships, and perceived stress and control; supportive non-family relationships did not remain statistically significant after controlling for other mediators.

**Conclusions:**

Findings suggest caregiver adaptation to a dystrophinopathy diagnosis can be optimized by increased perceived control, supporting family resources, and creation of a healthy family identity. Our findings will help identify areas for family intervention and guide clinicians in identifying resources that minimize stress and maximize family adaptation.

## Background

Duchenne (DMD) and Becker (BMD) muscular dystrophies, collectively termed dystrophinopathies, are X-linked disorders characterized by progressive muscle weakness [1]. Dystrophinopathies affect an estimated 2 per 10,000 boys [2–4] and are caused by abnormal dystrophin protein in the muscle [5]. Dystrophin is essentially absent in patients with DMD, whereas it is typically decreased in quantity or size in patients with the milder BMD phenotype. Typically, symptom onset for DMD occurs before the 5^th^ birthday and historically, complete loss of ambulation occurs by the 12^th^ birthday [6]. Symptom onset for BMD often occurs at a later age and disease progression is slower. Those affected by a dystrophinopathy experience progressive weakness resulting in loss of ability to walk or perform activities of daily living (ADLs). Compromised pulmonary and cardiac systems are the major contributors to premature mortality.

Treatment of dystrophinopathies with corticosteroids and aggressive pulmonary and cardiac management have decelerated loss of function and extended life expectancy [7–11]. Despite optimal treatment, loss of independence and need for assistance with ADLs remain inevitable [8, 12]; family members (usually parents) typically provide the majority of the care. In addition to caring for a child with significant weakness, these caregivers must cope with the additional psychological and physical co-morbidities associated with dystrophinopathies [13–15]. The associations between a dystrophinopathy diagnosis and poorer health-related quality of life of patients [16] and maladaptation of individual family members [12, 13, 17–21] are well documented. To our knowledge, disease impact on family quality of life (FQoL) has received less attention.

**Fig. 1:**
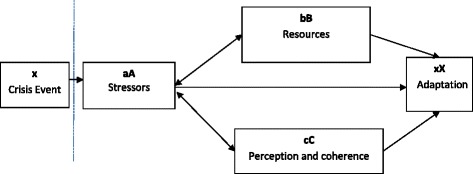
Double ABCX model, adapted from Lavee, McCubbin, & Patterson (1985)

The Double ABCX model of family stress and adaptation frequently has been used to examine processes that influence family adaptation to a crisis event (x) (Fig. 1; [22]). Stressor pile-up (aA) represents the cumulative demands over time that may arise after experiencing a crisis event. Intermediate factors that may affect the impact of stress on family adaptation include family adaptive resources (bB) and perception and coherence (cC). Adaptive resources may be comprised of personal resources or individual characteristics, family system attributes, and social support. Perception and coherence represents the family’s response and orientation to the stressor, which includes perceived predictability of the crisis event and the ability to handle the consequences of such events. Family adaptation (xX) is a measure of the family’s adjustment to an event.

The Double ABCX model has been used to study family adaption to chronic health conditions. The calculation of stressor pile-up has varied between studies with some studies using a count of recent stressful life events [23–26], whereas others used perceived caregiving burden [12, 15, 21] or child characteristics (e.g., age, adaptive skills, challenging behavior, level of disability) [27, 28] as indicators of stressor pile-up. Operationalizing family adaptive resources and perception and coherence has also varied across studies and included measures of family support, coping, or reframing [13, 15, 17, 18, 20, 23–30]. Family adaptation has been evaluated using a variety of outcomes including individual family dynamics or quality of life. We used the Double ABCX model to guide our retrospective analysis of associations between parental perceptions of resources available to manage a dystrophinopathy diagnosis and caregiver perceptions of FQoL using survey data collected from a cohort of caregivers of males with a diagnosis. Our findings will help guide clinicians and families in the evaluation of resources that may aid in minimizing this stress and maximizing the family’s ability to adapt to caring for an affected family member with a childhood-onset dystrophinopathy.

## Methods

The Muscular Dystrophy Surveillance, Tracking, and Research Network (MD STAR*net*) was established in 2002 by the Centers for Disease Control and Prevention to determine prevalence and track health services utilization and outcomes for childhood-onset dystrophinopathies in the United States [3, 31, 32]. In 2004, MD STAR*net* retrospectively identified and prospectively followed individuals born since January 1, 1982 who were diagnosed with a dystrophinopathy by age 21 years, and resided following diagnosis in an MD STAR*net* site (Arizona, Colorado, Iowa, western New York State). Georgia joined the MD STAR*net* in 2005 and Hawaii in 2008. A committee of neuromuscular clinical experts reviewed clinical and laboratory data to assign each cases identified a case definition (definite, probable, possible, asymptomatic, affected female, not affected) that reflected certainty of diagnosis using clinical signs and symptoms and available confirmatory biologic testing or maternal family history. Cases identified before September 2011 were followed through December 2011, and those identified after September 2011 were followed through December 2012. A primary caregiver of a male with a definite (confirmed by genetic testing, muscle biopsy, or creatine kinase testing with positive maternal family history) or probable (confirmed by maternal family history) dystrophinopathy diagnosis was eligible for participation (*n* = 460). The caregiver was asked to complete the mailed questionnaire for the oldest affected male living in the home; monetary compensation was provided. Institutional review board approval was obtained from each MD STAR*net* site.

### Caregiver questionnaire

The *Caregiver Questionnaire* was developed to evaluate caregiver perceptions of FQoL, social support, perceived stress and control, and spirituality, and collect data on caregiver sociodemographic characteristics, including race/ethnicity, marital status, education, and employment. Case characteristics included in the questionnaire were physical and mental health factors identified by MD STAR*net* clinicians as potential co-morbid conditions diagnosed among those affected by a dystrophinopathy that may be due to underlying disease expression or as complications of disease progression (e.g., restriction to a wheelchair), as well as current status of upper and lower extremity function as a measure of disease progression. Instruments used to measure these factors are summarized briefly below.

#### Stressor pile-up (aA factor)

Stressor pile-up includes caregiver responses to questions about: 1) presence of case mental health diagnoses (attention-deficit disorder, mental retardation, depression, anxiety, behavioral or conduct problems, developmental delay, autism, obsessive-compulsive disorder, schizophrenia, personality disorder); 2) presence of physical comorbidities (high blood pressure, cataracts, asthma, cerebral palsy, inflammatory bowel disease, migraine headaches, seizures, diabetes, gastroesophageal reflux, gallstones, kidney stones, deep vein thrombosis or blood clots, failure to thrive in obesity or later trouble gaining weight, obesity, cancer, pseudotumor cerebri, constipation, trouble urinating, and trouble holding urine); 3) scores on the clinically validated 6-point Brookes scale of upper extremity function [33] and 10-point Vignos scale of lower extremity function [34]; 4) social network stress scores as calculated for the stressfulness of 10 relationships types (e.g., spouse, parent) using the Duke Social Stress and Support Scale (DUSOCS) scoring instructions [35]; and 5) the presence of select demographics that are typically considered as barriers in social determinants of health.

The stressor pile-up count was based on the summing of the following 8 dichotomized (yes/no) indicators: 1) cases with two or more mental health diagnoses (n[yes] = 43, 22%); 2) cases with two or more physical health conditions (n[yes] = 86, 44%); 3) cases with low functional status (the inability to bring hands to mouth (Brookes Scale 6/6) and cannot walk even with assistance (Vignos scale > =8/10) [n[yes] = 68, 34%]); 4) caregivers’ high social stress (upper tertile of DUSOCS calculated stressful relationships distribution) (n[yes] = 71, 36%); 5) caregivers’ unmarried status (n[yes] = 45, 23%), 6) caregivers’ minority race/ethnicity (n[yes] = 37, 19%); 7) caregivers’ non-high school education attainment (n[yes] = 49, 25%); and 8) caregivers’ unemployment (n[yes] = 98, 50%).

#### Family adaptive resources (bB factor)

The Multidimensional Scale of Perceived Social Support (MSPSS) measures perceived availability of support and consists of 12 items rated on a 7-point Likert Scale (1 = Very strongly disagree; 7 = Very strongly agree) [36, 37]. Items were summed with higher scores representing greater perceived support availability. A high Cronbach’s alpha (α = 0.95) was observed for our summed score.

A supportive social network was also included as an adaptive resource by using the summed support score from the DUSOCs [35]. The caregiver rated the supportiveness of 10 relationships types (e.g., spouse, parent) using a 3-point Likert scale (0 = None, 1 = Some, 2 = A lot). Scores were calculated according to DUSOCs scoring instructions and ranged from 0 to 100. High family (DUSOCS-F) and non-family (DUSOCS-NF) DUSOCs supportive relationship scores represented potential sources of social support.

#### Family coherence (cC factor)

The 10-item Perceived Stress Scale (PSS10) measures appraisals of the caregiver stress level, including feelings of unpredictability, uncontrollability, and being overloaded by life situations [38, 39]. Caregivers rated how often they had such feelings using a 5-point Likert scale (0 = Never, 4 = Very often). Scores are summed with higher scores representing lower perceptions of controllability. The Cronbach’s alpha (α = 0.87) for our summed score was good.

The Functional Assessment of Chronic Illness Therapy Spiritual Well-Being Scale (modified) (FACIT-Sp) measures spiritual components of well-being (i.e., peacefulness, meaning and purpose, comfort from faith) [40, 41]. The questionnaire consists of 12-items on a 5-point Likert scale (1 = Not at all; 5 = Very much). Higher summed scores represent a greater sense of overall spiritual well-being. The Cronbach’s alpha (α = 0.88) for our summed score was good.

#### Family adaptation (xX factor)

The Beach Center Family Quality of Life Scale (FQoL) measures perceived family quality of life [42]. Caregivers rate the level of family satisfaction with available resources, supportive familial relationships, family adaptability, and access to needed resources. Twenty-five items were rated using a 5-point Likert scale (1 = Very dissatisfied, 2 = Dissatisfied, 3 = Neither, 4 = Satisfied, 5 = Very satisfied). Higher summed scores represent better perceptions of familial quality of life. Our observed Cronbach’s alpha for our total FQoL was high (α = 0.94).

### Statistical analyses

Participation rates were calculated using the American Association for Public Opinion Research calculator [43], which adjusts rates for those of unknown eligibility due to unconfirmed residence. The calculations produced from the calculator will differ slightly from observed counts that do not make this adjustment. To evaluate sample representativeness, characteristics of all eligible MD STAR*net* cases and caregivers were compared to those of the respondents. Next, each measure listed above was evaluated for item missingness. Multiple imputation was performed for measures with less than 20% missingness. Descriptive statistics (means [*M*], standard deviations [*SD*], counts, percentages) were calculated for continuous and categorical variables. To test for mediation, direct and indirect effects were computed using a series of ordinary least squares (OLS) regressions and a bootstrapping procedure recommended by Preacher and Hayes [44, 45]. An indirect effect represents the amount of reduction in the direct effect an independent variable has on the dependent variable after a mediator is introduced into the model. Single and multiple mediator models were run. The single mediator model evaluated indirect effects corresponding to each mediator independently. The multiple mediator model estimated indirect effects for each mediator with all variables entered simultaneously. The proportion of the total effect attributable to indirect effect(s) was also calculated using methods of Alwin and Hauser [46]. Statistical significance was set at *p* = 0.05 for bivariate correlational analyses; significance of indirect effects was determined by 95% confidence intervals (CI). SAS® software, Version 9.4 was used for analyses [Copyright (c) 2002-2012 by SAS Institute Inc., Cary, NC, USA.].

## Results

**Fig. 2:**
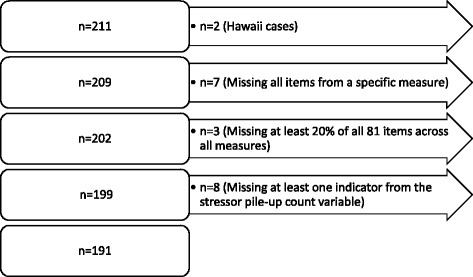
Case exclusions from analysis of the MD STARnet Caregiver Questionnaire

Questionnaires were completed by 211 primary caregivers from August 2011 through February 2012 (Fig. 2). We estimated a 51% response rate among all eligible caregivers, a 63% cooperation rate among those caregivers with known contact, and a 29% refusal rate among all caregivers [43]. Questionnaires (*n* = 2) completed by caregivers from the Hawaii MD STAR*net* site were excluded due to a reduced time frame for recruitment in survey research. Tests of sample representativeness showed respondents were more educated than non-respondents (Table 1).

**Table 1:** Comparison of eligible and responding families from the MD STAR*net*

Characteristic^a^	Eligible Families (*n* = 460)^b^		Responding Families (*n* = 209)^b^		*χ* ^2^ prob.
	No.	Percent	No.	Percent	
Case Status					0.59
Definite	448	97.4	205	98.1	
Probable	12	2.6	4	1.9	
Site					
Arizona	119	25.9	52	24.9	0.21
Colorado	101	22.0	36	17.2	
Georgia	119	25.9	49	23.4	
Iowa	69	15.0	45	21.5	
New York	52	11.3	27	12.9	
Child Year of Birth					0.66
1982–1985	28	6.1	16	7.7	
1986–1990	82	17.8	39	18.7	
1991–1995	120	26.1	64	30.6	
1996–2000	124	27.0	47	22.5	
2001–2005	81	17.6	32	15.3	
≥ 2006	25	5.4	11	5.3	
Caregiver Relationship					0.42
Biologic Mother	405	88.0	193	92.4	
Biologic Father	23	5.0	6	2.9	
Adoptive	14	3.0	6	2.9	
Foster	2	0.4	1	0.5	
Other	16	3.5	3	1.4	
Maternal Age at Questionnaire Completion^c^					0.75
< 30	14	4.8	4	3.0	
30–34	32	11.0	15	11.1	
35–39	58	19.9	22	16.3	
40–44	71	24.4	33	24.4	
45–49	67	23.0	39	28.9	
≥ 50	49	16.8	22	16.3	
Missing	(169)		(74)		
Maternal Race/Ethnicity					0.14
Non-Hispanic Black	26	6.7	9	5.1	
Non-Hispanic White	270	69.1	139	78.5	
Hispanic or Latino/Latina	78	20.0	24	13.6	
Other^5^	17	4.3	5	2.8	
Missing	(69)		(32)		
Maternal Education					0.04
12^th^ grade or less, no diploma	81	24.1	22	14.0	
High School graduate or GED	103	30.7	46	29.3	
Some college or 2-year degree	73	21.7	41	26.1	
Bachelor’s degree or higher	79	23.5	48	30.6	
Missing	(124)		(52)		
Paternal Age at Questionnaire Completion^c^					0.80
< 30	5	2.0	1	0.9	
30–34	20	8.1	10	8.6	
35–39	43	17.5	16	13.7	
40–44	51	20.7	22	18.8	
45–49	54	22.0	31	26.5	
≥ 50	73	29.7	37	31.6	
Missing	(214)		(92)		
Paternal Race/Ethnicity					0.05
Non-Hispanic Black	22	6.5	8	5.1	
Non-Hispanic White	247	72.9	130	81.8	
Hispanic or Latino/Latina	59	17.4	21	13.2	
Other^d^	11	3.2	0	0.0	
Missing	(121)		(50)		
Paternal Education					0.23
12^th^ grade or less, no diploma	47	16.9	13	9.9	
High School graduate or GED	89	32.0	43	32.6	
Some college or 2-year degree	69	24.8	33	25.0	
Bachelor’s degree or higher	73	26.3	43	32.6	
Missing	(182)		(77)		

*No.* number, MD STAR*net* Muscular Dystrophy Surveillance, Tracking, and Research Network. Missing values were not included in chi-square analyses

^a^Characteristics, for example, site, were obtained from the latest surveillance data (v8). Such values may differ from those recorded at questionnaire completion. Maternal and paternal race/ethnicity information was obtained from the respective calculated variables

^b^Eligible = Families with a case classification of “probable” or “definite”, excluding those from Hawaii, who were eligible for the Caregiver questionnaire. Respondent = completed questionnaire received between August 2011 and February 2012

^c^Maternal and paternal ages at questionnaire completion for non-respondents were calculated as the “mid-point year from completed questionnaires” (2012) – “year of birth”

^d^Other race/ethnicity includes Asian or Hawaiian or Pacific Islander, Native American or American Indian or Alaska Native, multiple and other unclassified types, excluding unknown

After handling missing data, our final analytic dataset comprised 191 caregivers. Mean caregiver age at questionnaire completion was 45.1 years (*SD* = 8.8) and the majority of the respondents were the biologic mother (92%) (data not shown). Most caregivers (78%) were married or living as married; 83% were non-Hispanic white; 50% were employed full-time; and 83% had completed some college or a higher degree. Mean age of cases at time of questionnaire completion was 16.5 years (*SD* = 6.1).

### Mediation analyses

#### Single mediator models

Correlational analyses showed significant bivariate associations between all variables and stressor pile-up, except spirituality (Table 2). Results for each single mediator model were consistent with partial mediation (Table 3) [44, 47]. Specifically, the total direct effect of stressor pile-up on FQoL was statistically significant. The direct pathways between stressor pile-up and FQoL remained significant, albeit reduced in magnitude, after entering each single mediator into the model (Table 3). Higher stressor pile-up was associated with lower perceived FQoL when summed scores for perceived resources were lower (MSPSS, DUSOCS-F, DUSOCS-NF) and those for perceived stress and lack of control (PSS) were higher. The proportion explained of the total effect (PE) ranged from 13% for a supportive non-family social network to approximately 50% for each of the remaining mediators. Less than 40% of the variance in FQoL was explained by each of the individual mediation models (Table 3).

**Table 2:** Pearson-moment correlations between study variables from caregiver responses to the MD STAR*net Caregiver Questionnaire* (*n* = 191)^1^

Variables	Stressor Pile-up	MSPSS	DUSOCS-F	DUSOCS-NF	PSS	FACIT-Sp	FQoL
Stressor Pile-up (aA)							
Family Resources (bB)							
Perceived Social Support (MSPSS)	−0.29^c^						
Supportive Relationships: Family (DUSOCS-F)	−0.30^c^	0.53^c^					
Supportive Relationships: Non-Family (DUSOCS-NF)	−0.19^b^	0.37^c^	0.25^b^				
Perception and Coherence (cC)							
Perceived Stress (PSS)	0.33^c^	−0.44^c^	−0.30^c^	−0.32^c^			
Spirituality (FACIT-Sp)	−0.14	0.27^b^	0.27^b^	0.31^c^	−0.56^c^		
Family Quality of Life (FQoL) (xX)	−0.29^b^	0.58^c^	0.52^c^	0.25^b^	−0.49^c^	0.51^c^	
Mean	2.49	66.57	57.67	37.17	16.64	33.72	105.19
SD	1.45	14.09	23.72	24.31	6.44	9.12	13.52
Median	2.00	66.00	57.14	40.00	17.00	35.00	105.00
Min, max	0, 6	12, 84	0, 100	0, 100	1, 31	8, 48	61, 125

*SD* Standard deviation, *Min* minimum score, *Max* maximum score, MD STAR*net* Muscular Dystrophy Surveillance, Tracking and Research Network, *MSPSS* Multidimensional Scale of Perceived Social Support, *DUSOCS-F* Duke Social Stress and Support Scale – Family, *DUSOCS-NF* Duke Social Stress and Support Scale – non-Family, *PSS* Perceived Stress Support, *FACIT-SP* Functional Assessment of Chronic Illness Therapy 12-item Spiritual Well-Being Scale (modified), *FQoL* Beach Center Family Quality of Life Scale

^a^ *p* < 0.05. ^b^ *p* < 0.01. ^c^ *p* < 0.001

^1^Questionnaires completed August 2011 through February 2012

**Table 3:** Single mediator models predicting FQoL from caregiver responses to the MD STAR*net Caregiver Questionnaire* (*n* = 191)^1^

Mediation Models	Total Effect	Direct Effect (Path c’)				Mediator to DV (Path b)				Indirect Effect				Proportion
	(Path c)	b	SE	95% CI		b	SE	95% CI		b	SE	95% CI		Total Effect
Total effect Stressor pile-up on FQoL	−2.75													
Model 1: MSPSS^2^														
Stressor-pileup		−1.28	0.57	−2.40	−0.15									
MSPSS						0.52	0.06	0.41	0.64	−1.47	0.46	−2.54	−0.67	53%
Model 2: PSS^3^														
Stressor-pileup		−1.38	0.62	−2.61	−0.15									
PSS						−0.92	0.14	−1.20	−0.65	−1.37	0.38	−2.19	−0.77	50%
Model 3: DUSOCS-F^4^														
Stressor-pileup		−1.42	0.60	−2.61	−0.23									
DUSOCS-F						0.27	0.04	0.20	0.34	−1.33	0.32	−1.98	−0.74	48%
Model 4: DUSOCS-NF^5^														
Stressor-pileup		−2.40	0.65	−3.68	−1.12									
DUSOCS-NF						0.11	0.04	0.03	0.19	−0.35	0.21	−0.98	−0.07	13%

*DV* dependent variable, *b* unstandardized regression coefficient, *SE* standard error, *CI* confidence interval, *FQoL* Family Quality of Life, MD STAR*net* Muscular Dystrophy Surveillance, Tracking and Research Network, *MSPSS* Multidimensional Scale of Perceived Social Support, *DUSOCS-F* Duke Social Stress and Support Scale – Family, *DUSOCS-NF* Duke Social Stress and Support Scale – non-Family, *PSS* Perceived Stress Support, *FACIT-SP* Functional Assessment of Chronic Illness Therapy 12-item Spiritual Well-Being Scale (modified), *FQoL* Beach Center Family Quality of Life Scale

*Note*: The proportion explained in the total effect by the indirect effect = indirect effect/total effect

^1^Questionnaires completed August 2011 through February 2012. ^2^ R^2^ = 0.36, F(2188) = 52.10, *p* < .001. ^3^ R^2^ = 0.26, F(2188) = 32.62, *p* < .001. ^4^ R^2^ = 0.29, F(2188) = 38.71, *p* < .001. ^5^ R^2^ = 0.12, F(2188) = 13.30, *p* < .001

#### Multiple mediator models

**Fig. 3:**
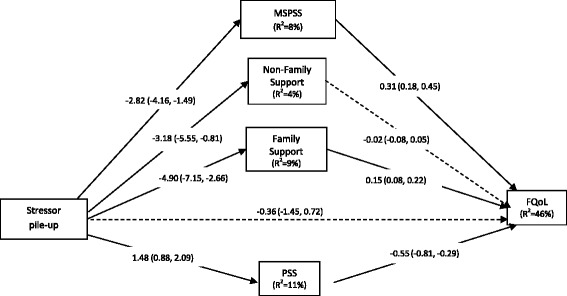
Double ABCX multiple mediator model from caregiver responses to the MD STARnet Caregiver Questionnaire (*n* = 191)^1^. *Abbreviations*: *DV* dependent variable, *b* unstandardized regression coefficient, *SE* standard error, *R* ^*2*^ proportion variance explained, *CI* confidence interval, *MSPSS* Multidimensional Scale of Perceived Social Support, *DUSOCS-F* Duke Social Stress and Support Scale – Family, *DUSOCS-NF* Duke Social Stress and Support Scale – non-Family, *PSS* Perceived Stress Support, *FACIT-SP* Functional Assessment of Chronic Illness Therapy 12-item Spiritual Well-Being Scale (modified), *FQoL* Beach Center Family Quality of Life Scale. *Note*: Multiple mediator model (Model 4) takes into account correlations between mediators in predicting FQoL; pathways from stressor to mediator are equivalent to the respective bivariate associations. Dashed line=statistically non-significant; Solid line=statistically significant. ^1^Questionnaires completed August 2011 through February 2012

Results for the multiple mediator model [45] showed multiple pathways through which high stressor pile-up was associated with lower perceived FQoL (Table 4 and Fig. 3). Higher stressor pile-up was associated with lower MSPSS, DUSOCS-F, and higher PSS. In turn, each of these were associated with FQoL. The pathway for DUSOCS-NF did not remain statistically significant after controlling for all other pathways. Each significant pathway accounted for approximately one-third of the total effect (Table 4). Nearly one-half of the variance in FQoL was explained by the multiple mediator model (R^2^ = 0.46; F(5, 185) = 31.39, *p* < 0.001).

**Table 4:** Multiple mediator model predicting FQoL from caregiver responses to the MD STAR*net Caregiver Questionnaire* (n = 191)^1^

Variables (Effects)	Total Effect	Direct effect (Path c’)				Mediator to DV (Path b)				Indirect Effect				Proportion
	(Path c)	b	SE	95% CL		b	SE	95% CL		effect	SE	95% CL		Total Effect
Total effect: stressor pile-up on FQoL	−2.84													
Mediation model:														
Stressor-pileup (Direct)		−0.36	0.55	−1.45	0.72									13%
MSPSS (Indirect)						0.31	0.07	0.18	0.45	−0.89	0.41	−1.82	−0.24	31%
PSS (Indirect)						−0.55	0.13	−0.81	−0.29	−0.81	0.28	−1.46	−0.38	29%
DUSOCS-Family (Indirect)						0.15	0.04	0.08	0.22	−0.73	0.26	−1.41	−0.32	26%
DUSOCS-Non-family (Indirect)						−0.02	0.03	−0.08	0.05	0.05	0.15	−0.26	0.35	2%

*DV* dependent variable, *b* unstandardized regression coefficient, *SE* standard error, *CI* confidence interval, *MSPSS* Multidimensional Scale of Perceived Social Support, *DUSOCS-F* Duke Social Stress and Support Scale – Family, *DUSOCS-NF* Duke Social Stress and Support Scale – non-Family, *PSS* Perceived Stress Support, *FACIT-SP* Functional Assessment of Chronic Illness Therapy 12-item Spiritual Well-Being Scale (modified), *FQoL* Beach Center Family Quality of Life Scale

*Note*: The proportion explained in the total effect by the indirect effect = indirect effect/total effect

^1^Questionnaires completed August 2011 through February 2012

## Discussion

We used the Double ABCX model as a theoretical model to guide analyses of associations between stressor pile-up, family resources, and FQoL among families affected by a childhood-onset dystrophinopathy. Stressor pile-up was comprised of disease-related indicators (e.g., comorbid mental and physical health conditions, reduced functional status), social network stress, and sociodemographic characteristics (e.g., education, race/ethnicity). Our results were consistent with previous studies of chronic childhood diseases showing inverse associations between high stressor pile-up and family adaptation, and a reduction of this association by adequate social support and perceived manageability of stress [15, 17, 23, 26–29, 48, 49]. Our results also highlight the resiliency of these families in response to stressors. Specifically, the average scores for caregiver reports on perceived FQOL were towards the high end of the distribution. Caregiver perceptions of available social support and spirituality were also near the high end and perceived unmanageability of stress were towards the low end of their respective distributions. These findings support the proposition that, in the presence of significant risk exposure, the potential for families to demonstrate resiliency is increased when existing resources are available and sufficient to respond to a crisis event [50].

Family stress theory describes processes involved in balancing family demands with family capabilities to adapt to such demands [50]. Family adaptation is conceptualized as resulting from the capabilities of families or individual family members to utilize resources in response to demands. From this response, the family is able to assign meaning to their situation, develop a family identity separate from the diagnosis, and establish relationships with supportive extra-familial environments [51]. In our study, caregiver respondents were predominantly non-Hispanic white and had at least some college education; thus, financial resources available to the family may have protected against some effects of stress on family adaptation. Additionally, the association between sufficient resources, such as social support, which has long been viewed as an important factor in reducing the effect of stress on adaptation [52], and healthy family adaptation is consistent with findings from studies of parents of children with special needs [15, 17–19, 27, 28, 53–56]. Parental cognitions, such as perception of an event as predictable and controllable, may also minimize the impact of stress on adaptation by empowering the family unit to cope with demands [57]. High perceived stress has been shown to negatively influence family adaptation to chronic childhood disease [17, 18, 20, 24, 55, 58] and contribute to increased negative perceptions of stressful situations, perceived manageability, and meaningfulness of life [28]. In our study, caregivers who reported their recent stress as more manageable also reported higher FQoL. Finally, religious coping has been found to predict better well-being in some [59, 60], but not all studies [61]. We observed higher spiritual well-being was not associated with stressor pile-up, but was associated with higher FQoL, which has been reported previously [62].

From the family stress perspective, healthy adaptation to a progressive disease will involve promoting utilization of resources (existing and new), assisting with developing a family identity, and promoting relationships outside of the family environment for all family members. During a workshop (*Facilitating family adjustment to a diagnosis of Duchenne muscular dystrophy*) sponsored by the Parent Project Muscular Dystrophy [63], factors that may impact family adjustment to a dystrophinopathy diagnosis were identified and recommendations for promoting healthy adaptation by all members of the family were made. Similar recommendations were incorporated into the care recommendations for patients with DMD [2]. Central to these recommendations is the optimization of quality of life by making information about the disease accessible and promoting appropriate care that adequately manages primary and comorbid conditions. Access to information and the provision of appropriate care should promote a patient’s and family’s sense of predictability and confidence in management of this progressive and variable disease, as well as provide the patient with adaptive resources that would ensure continued participation of the patient in the family and community. Using formal (e.g., mental health professionals) and informal (e.g., parent) supportive networks was also encouraged along with the provision of resources for identifying sources of financial support and assistance with respite care options. Each of these recommendations could contribute to healthy family adaptation by promoting a perception of control over the impact of the disease, establishing resources within and outside of the family, and creating a family identity that encourages a perception of empowerment over healthy adaptation to current and future stressors.

Strengths of our study include the recruitment of care providers from a population-based sample of families managing a childhood-onset dystrophinopathy diagnosis [32], which allowed evaluation of sample representativeness. Simultaneous inclusion of multiple measures of adaptation and factors that may affect adaptation (e.g., sociodemographic variables) into our analytic models allowed a comprehensive evaluation of resources that promote family resilience to a chronic health condition [50]. Family adaptation to a chronic disease may vary by severity of disease expression. Greater adaptation may be observed among families of children with less severe presentation (i.e., BMD) due to fewer challenges to family resources. The inclusion of disease characteristics as a component of stressor pile-up takes into account disease severity (e.g., DMD versus BMD) by counting loss of functioning of upper or lower extremities as potential contributors to stressor pile-up.

Several limitations from our study should also be recognized. Analysis of sample representativeness showed respondents to be more highly educated than the general MD STAR*net* population possibly limiting generalizability of findings to families with less educated caregivers. Also, caregivers, most often the mother, reported on all measures included in the questionnaire, which might result in a common method variance due to single source bias and inflate correlations between measures. Relatedly, multiple respondents from each family were not considered in the protocol, thereby precluding any comparison of individual perceptions in any one family [42]. Stressors may differentially influence individual family members as observed in previous evaluations of both maternal and paternal perceptions [28, 56, 64, 65]. Another limitation is that information about specific coping strategies (e.g., problem-focused, active avoidance) that might be considered a resource when managing stress was not collected. Previous studies of family adaptation to autism spectrum disorder have shown maladaptive coping strategies (e.g., avoidance and disorganization) are associated with poorer family outcomes [23, 25, 56]. Lastly, our study used a cross-sectional design, precluding evaluation of time ordering of measures included in the model, and evaluation of causality; however, most of the components of the indicator for stressor pile-up would not be responsive to other individual or family characteristics (e.g., functional ability and co-morbid conditions of the case), which justifies modeling pile-up as a causal factor. Further, although dystrophinopathies are chronic diseases to which families may show greater adaptation as time passes, childhood-onset dystrophinopathies have an evolving presentation with the emergence of new morbidities (e.g., loss of mobility, pulmonary and cardiac dysfunction). This requires continuous adaption by the family over time. As a cross-sectional study, the questionnaires did not collect information about timing of such morbidities, as such, time since diagnosis was not evaluated. Thus, it is also crucial that further detailed investigations are necessary using a longitudinal design where comprehensive clinical and family information is collected prospectively on large, multi-center samples using rigorous analyses to develop a better understanding of the main, as well as moderating and mediating, effects of multiple levels of factors on family quality of life. This is a necessary step before considering research to identify specific interventions.

## Conclusions

Although the Double ABCX model has been used to describe functioning of families affected by chronic childhood diseases [23, 27–29], to our knowledge, the model has not been applied within the context of childhood-onset dystrophinopathies, which are progressive and terminal, nor has FQoL been examined as the indicator of family functioning within this context. Our findings contribute to the literature on family adaption to chronic disease by describing functioning of families affected by child-onset dystrophinopathies and identifying potential areas for family intervention that could promote resiliency among those struggling with management of these diseases. Future research should incorporate prospective, longitudinal studies to further delineate those qualities that contribute to family adaption to a dystrophinopathy diagnosis so that specific interventions that promote these qualities can be implemented.

## Abbreviations

aA: stressor pile-up
ADL: Activities of daily living
b: unstandardized regression coefficients
bB: family adaptive resources
BMD: Becker muscular dystrophy
cC: perception and coherence
CI: Confidence intervals
DMD: Duchenne muscular dystrophy
DUSOCS: Duke Social Stress and Support Scale
DUSOCS-F: Duke Social Stress and Support Scale – Family
DUSOCS-NF: Duke Social Stress and Support Scale – non-Family
DV: Dependent variable
FACIT-Sp: Functional Assessment of Chronic Illness Therapy Spiritual Well-Being Scale (modified)
FARA: Friedreich ataxia research alliance
FQoL: Family quality of life
IV: Independent variable
M: Means
Max: Maximum score
MD STARnet: Muscular Dystrophy Surveillance, Tracking, and Research Network
Min: Minimum score
MSPSS: Multidimensional Scale of Perceived Social Support
No.: Number
OLS: Ordinary least squares
PE: Proportion explained of the total effect
PSS: Perceived stress and lack of control
PSS10: 10-item Perceived Stress Scale
R^2^: Proportion variance explained
SD: Standard deviations
SE: Standard error
X: Crisis event
xX: Family adaptation
